# Perinatal outcomes following single intrauterine death in monochorionic twin pregnancies complicated by twin anemia polycythemia sequence: Systematic review and meta‐analysis

**DOI:** 10.1111/aogs.70189

**Published:** 2026-05-09

**Authors:** Marina Piergianni, Asma Khalil, Giuseppe Rizzo, Ilenia Mappa, Lorenza Della Valle, Hiba J. Mustafa, Alberto Galindo, Alireza A. Shamshisaz, Francesco D'Antonio

**Affiliations:** ^1^ Center for Fetal Care and High‐Risk Pregnancy University of Chieti Chieti Italy; ^2^ Fetal Medicine Unit St George's University Hospital London UK; ^3^ Vascular Biology Research Centre, Molecular and Clinical Sciences Research Institute St George's University of London London UK; ^4^ Fetal Medicine Unit, Liverpool Women's Hospital University of Liverpool Liverpool UK; ^5^ Department of Maternal and Child Health and Urological Sciences Sapienza University of Rome Rome Italy; ^6^ Division of Maternal‐Fetal Medicine, The Fetal Center at Riley Children's and Indiana University Health, Indiana University School of Medicine Riley Children's Hospital Indianapolis Indiana USA; ^7^ Fetal Medicine Unit, Department of Obstetrics and Gynecology, Hospital Universitario 12 de Octubre Complutense University Madrid Spain; ^8^ Instituto de Investigación del Hospital 12 de Octubre (imas12) Madrid Spain; ^9^ Primary Care Interventions to Prevent Maternal and Child Chronic Diseases of Perinatal and Developmental Origin (RICORS Network) Madrid Spain; ^10^ Division of Maternal Fetal Medicine, Department of Obstetrics and Gynecology Beth Israel Deaconess Medical Center Boston Massachusetts USA; ^11^ Division of Fetal Medicine and Surgery, Department of Surgery Boston Children's Hospital Boston Massachusetts USA; ^12^ Department of Obstetrics, Gynecology, and Reproductive Sciences Harvard Medical School Boston Massachusetts USA

**Keywords:** brain, death, IUFD, meta‐analysis, neurological, outcome, preterm, systematic review, TAPS, twin pregnancy

## Abstract

**Introduction:**

Most of the studies reporting the outcome of the surviving twin after single intra‐uterine fetal death (sIUFD) focused on uncomplicated pregnancies or those affected by twin‐to‐twin transfusion syndrome, while there is a paucity of data for those complicated by twin anemia polycythemia sequence (TAPS). The aim of this systematic review was to report perinatal outcomes in monochorionic diamniotic (MCDA) twin pregnancies complicated by TAPS after sIUFD, according to gestational age at fetal loss and the presence of pregnancy‐related comorbidities.

**Material and Methods:**

MEDLINE, EMBASE, and The Cochrane Library were searched for studies reporting the outcome of monochorionic twin pregnancies complicated by TAPS experiencing single IUFD. The primary outcome was the occurrence of co‐twin IUFD. Secondary outcomes were neonatal death (NND), preterm birth (PTB) <34, <32, and <28 weeks of gestation, cerebral abnormalities detected at follow‐up prenatal ultrasound, fetal magnetic resonance imaging (MRI) or postnatal imaging (ultrasound or MRI), and adverse neurodevelopmental outcome. Random‐effects meta‐analysis of proportions was used to analyze the data, and results were reported as pooled proportions or odd ratios (OR) with 95% CI.

**Results:**

Eleven studies (83 twin pregnancies affected by TAPS, either spontaneous or post‐laser, and complicated by single IUFD) were included in the systematic review and 10 (78 pregnancies) in the meta‐analysis. Co‐twin IUFD occurred in 4.9% (95% CI 1.1–11.3) after a single IUFD in pregnancies affected by TAPS, while there was no case of NND. PTB, either spontaneous or iatrogenic, occurred in 80.4% (95% CI 44.3–99.5) <34 weeks, and 50.0% (95% CI 16.3–83.7) <28 weeks of cases. Intra‐uterine transfusion was required in 8.0% (95% CI 0.9–38.3). Cerebral anomalies at follow‐up ultrasound or fetal MRI were reported in 15.0% (95% CI 8.2–78.3) and 11.4% (95% CI 2.4–55.7) of cases, while anomalies at post‐natal imaging in 9.4% (95% CI 5.6–57.6) of cases. It was not possible to perform a meaningful pooled data synthesis on the observed outcomes according to the type and staging of TAPS and according to the donor or recipient twin.

**Conclusions:**

The occurrence of co‐twin death after single IUFD in pregnancies complicated by TAPS appears low, while cerebral abnormalities detected either pre‐ or post‐natally occur in 10–15% of cases. Further evidence is needed to elucidate the long‐term neurological and neurodevelopmental risk of these children.

## Graphical abstract

The aim of this systematic review was to report perinatal outcomes in monochorionic diamniotic (MCDA) twin pregnancies complicated by twin anemia polycythemia sequence (TAPS) after single intrauterine fetal death (IUFD).

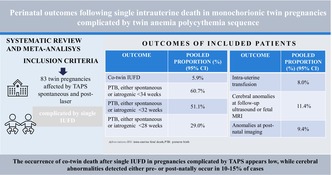

**Abbreviations**
MCA: middle cerebral artery
MCDA: monochorionic diamniotic
MoM: multiples of the median
MRI: magnetic resonance imaging
NND: neonatal death
NOS: Newcastle–Ottawa Scale
PSV: peak systolic velocity
PTB: preterm birth
sIUFD: single intra‐uterine fetal death
TAPS: twin anemia polycythemia sequence
TTTS: twin‐to‐twin transfusion syndrome

**Key message**

The occurrence of co‐twin death after single intra‐uterine fetal death in monochorionic diamniotic twin pregnancies complicated by twin anemia polycythemia sequence appears low, while cerebral abnormalities detected either pre‐ or post‐natally occur in 10%–15% of cases.

## 1 INTRODUCTION

Monochorionic diamniotic twin pregnancies (MCDA) are at higher risk of perinatal mortality and morbidity compared to dichorionic twin pregnancies, mainly due to preterm birth (PTB), fetal growth restriction, and complications unique to monochorionic placenta, such as twin‐to‐twin transfusion syndrome (TTTS) and twin anemia polycythemia sequence (TAPS).^1^, ^2^, ^3^, ^4^

The occurrence of single intra‐uterine fetal death (sIUFD) in a MCDA pregnancy can adversely affect the surviving twin, with a high risk of co‐twin IUFD, neonatal death (NND), preterm birth (PTB), and abnormal neurodevelopmental outcome.^5^, ^6^, ^7^ The pathophysiology of this phenomenon is probably related to acute hemorrhage from the surviving twin into the fetoplacental unit of the demised twin via the placental anastomoses, resulting in acute hypotension, cerebral ischemia, and long‐term neurological disability.^8^

However, most of the studies reporting the outcome of the surviving twin after sIUFD focused on uncomplicated pregnancies or those affected by TTTS, while there is a paucity of data for TAPS. TAPS occurs in 3–16% of MCDA pregnancies, either spontaneously or after laser surgery for TTTS and is caused by unbalanced slow transfusion of red blood cells through a few small (<1 mm diameter) placental arteriovenous anastomoses, resulting in anemia of one twin and polycythemia of the cotwin.^9^ The differences in placental angioarchitecture in TAPS compared to uncomplicated pregnancies or those affected by TTTS may account for a different risk of adverse outcome in the surviving twin after sIUFD.

In this context, we performed a systematic review and meta‐analysis to evaluate the perinatal outcomes after sIUFD in MCDA twin pregnancies affected by TAPS.

## 2 MATERIAL AND METHODS

### 2.1 Data sources

This review was performed according to an a priori‐designed protocol recommended for systematic reviews and meta‐analyses.^10^, ^11^ MEDLINE, EMBASE, and The Cochrane Library were searched electronically on 01/07/2025, utilizing combinations of the relevant medical subject heading (MeSH) terms, keywords, and word variants for “twin pregnancies”, “twin anemia polycythemia”, and “intra‐uterine death”. The search and selection criteria were restricted to the English language. Reference lists of relevant articles and reviews were searched manually for additional reports. Preferred Reporting Items for Systematic Reviews and Meta‐Analyses (PRISMA) guidelines were followed.^12^ The study was registered with the PROSPERO database (registration number: CRD42024543164).

### 2.2 Main outcomes and measures

Inclusion criteria were MCDA twin pregnancies affected by TAPS and complicated by sIUFD. TAPS was defined antenatally as the presence of a combination of middle cerebral artery (MCA) peak systolic velocity (PSV) ≥1.5 MoM in the anemic twin and ≤0.8 MoM in the polycythemic twin or, alternatively, as a MCA‐PSV discordance ≥1 MoM can be used to diagnose TAPS. ^13^ (Table S1).

The primary outcome was co‐twin IUFD, defined as loss of the surviving twin after the initial sIUFD event.

The secondary outcomes were:

- co‐twin NND, defined as death within 28 days after birth
- PTB <34, < 32, and <28 weeks of gestation, either spontaneous or iatrogenic; spontaneous PTB <34 weeks of gestation
- Need for fetal blood transfusion after IUFD for signs of fetal anemia in the donor, defined as middle cerebral artery (MCA) peak systolic velocity (PSV) >1.5 multiples of the median (MoM), cardiomegaly, ascites or hydrops
- cerebral anomalies detected at follow‐up fetal ultrasound or fetal MRI, including signs of peri‐ventricular or parenchymal ischemia or hemorrhage (any grade)
- cerebral anomalies detected at postnatal imaging (either cranial ultrasound or MRI), including signs of peri‐ventricular or parenchymal ischemia or hemorrhage (any grade)
- adverse neurodevelopmental outcome.

We planned to explore all outcomes in the overall population of MCDA twin pregnancies complicated by TAPS and sIUFD, according to the type (post‐laser vs. spontaneous) and staging (I–V) of TAPS separately.

### 2.3 Eligibility criteria

Two independent investigators (M.P, L.D.V.) examined the abstracts of all potentially relevant articles for suitability. Articles were excluded at this stage only if they did not meet the inclusion criteria. Full‐text copies of the remaining articles were obtained and assessed independently for content, data extraction, and analysis. Disagreements between the two investigators were resolved by discussion with a third author (F.D.A.). Study characteristics were extracted using a predesigned data extraction protocol. If more than one study was published on the same cohort with identical endpoints, only the report containing the most comprehensive information on the population was included to avoid overlapping populations.

We included studies reporting the outcome of MCDA twin pregnancies diagnosed with TAPS and complicated by sIUFD. Studies reporting exclusively on the outcome of the surviving twin after selective termination via cord occlusion were excluded because the acute interruption of blood flow in the umbilical cord following occlusion may not be representative of the natural history of IUFD. Likewise, we excluded cases with double IUFD and those in which chorionicity could not be determined or was not reported by the authors. Only full‐text articles were considered eligible for inclusion. Case reports, conference abstracts, and case series with fewer than two cases of IUFD were also excluded to avoid publication bias. We also excluded studies published before 2010 because we assume that advances in the prenatal management of TAPS made over that time render older studies less relevant.

The quality assessment of the included studies was carried out using the Newcastle–Ottawa Scale (NOS) for case–control and cohort studies.^14^ According to the NOS, each study is evaluated across three broad domains: selection of the study groups; comparability of the groups; and ascertainment of the outcome of interest. The assessment of the selection domain includes examining the representativeness of the exposed cohort, the selection of the non‐exposed cohort (in the case of a case–control study), the ascertainment of exposure, and demonstrating that the outcome of interest was not present at the start of the study. The assessment of the comparability domain involves evaluating the comparability of the cohorts based on the study's design or analysis. Lastly, the assessment of the outcome domain includes reviewing the type of outcome assessment and the duration and adequacy of follow‐up. According to the NOS, a study can be awarded a maximum of one star for each item within the selection and outcome domains, while a maximum of two stars can be given for the comparability domain.^15^

### 2.4 Data collection and analysis

Random‐effects meta‐analysis of proportions was employed to combine data.^16^ Funnel plots showing the outcome rate from individual studies *agains*t their precision (1/standard error) were used for exploratory purposes. Tests for funnel plot asymmetry were not performed when the total number of publications included for each outcome was fewer than 10. In such cases, the statistical power of these tests is too low to differentiate between chance and genuine asymmetry.^17^

Between‐study heterogeneity was examined using the *I* ^2^ statistic, which indicates the proportion of variation between studies attributable to heterogeneity rather than chance. A value of 0% signifies no observed heterogeneity, while *I* ^2^ values of ≥50% denote a substantial degree of heterogeneity.

Statistical analysis was performed using StatsDirect Statistical Software (StatsDirect Ltd., Cambridge, UK).

## 3 RESULTS

### 3.1 General characteristics of the studies

**FIGURE 1:**
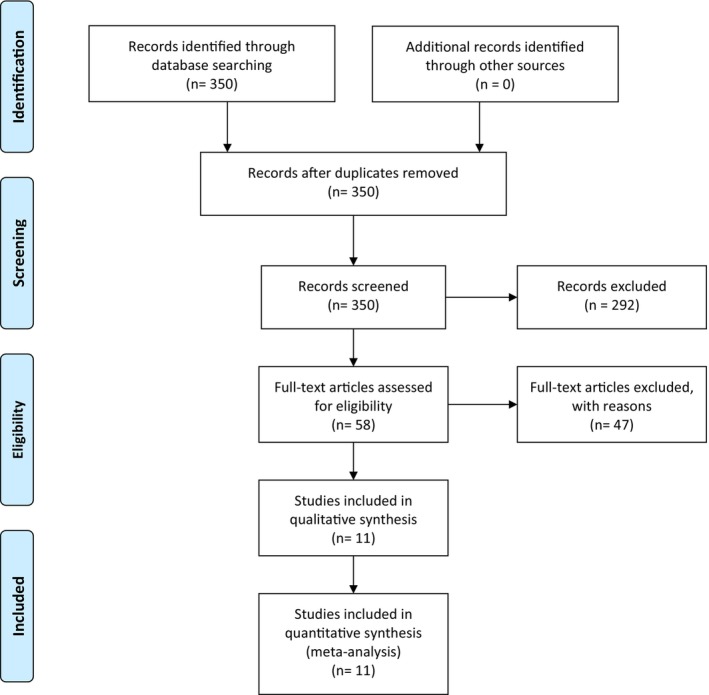
PRISMA flow diagram.

The literature search identified 350 articles, of which 11 were assessed for eligibility (Table 1, Figure 1, Table S2). After exclusions, 11 studies, comprising 83 twin pregnancies affected by TAPS and complicated by sIUFD, were included.^15^, ^18^, ^19^, ^20^, ^21^, ^22^, ^23^, ^24^, ^25^, ^26^, ^27^ The study by Mustafa et al. was not included in the pooled data synthesis because it included mostly cases with TTTS with signs of hemoglobin discordance, which may be erroneously termed as affected by TTTS+TAPS.

**TABLE 1:** General characteristics of studies included in systematic review and meta‐analysis.

Author	Year	Country	Study design	Period considered	Gestational age at IUFD	Type of TAPS	Prenatal imaging	Neurodevelopmental assessment	Timing at follow up (years)	Pregnancies (*n*)	IUFD (*n*)
Van De Sande^18^	2025	The Netherlands, Belgium, Italy, France, Canda, Argentina, Chile	Retrospective	2014–2023	23.0 (20.7–27.9)^a^	Spontaneous and post‐laser	US, MRI	Not reported	Not reported	432	39
Birk^19^	2025	United States	Retrospective	2014–2022	Not reported	Post‐laser	US	Not reported	Not reported	12	3
Tricca^20^	2024	Italy	Retrospective	2006–2023	Not reported	Spontaneous and post‐laser	US, MRI	Not reported	First month of life	29	14
Mustafa^21^	2022	United States	Retrospective	2012–2022	Not reported	Post‐laser	US	Not reported	Not reported	17	5
Rosen^22^	2022	Israel	Retrospective	2013–2021	Not reported	Spontaneous and post‐laser	US, MRI	Not reported	Not reported	23	4
Jeong^15^	2022	South Korea	Retrospective	2015–2019	Not reported	Post‐laser	US	Not reported	Not reported	10	2
Donepudi^23^	2016	United States	Retrospective	2011–2014	Not reported	Post‐laser	US	Not reported	Not reported	11	2
Fisher^24^	2016	Australia	Retrospective	2006–2014	Not reported	Spontaneous and post‐laser	US	Not reported	Not reported	10	2
Veujoz^25^	2015	France	Retrospective	2006–2013	Not reported	Spontaneous and post‐laser	US	Not reported	Not reported	20	2
Slaghekke^26^	2014	The Netherlands, France	Retrospective	2005–2013	Not reported	Spontaneous and post‐laser	US	Not reported	Not reported	52	7
Ruano^27^	2013	United States	Retrospective	2010–2012	Not reported	Post‐laser	US	Not reported	Not reported	6	3

Abbreviations: +US, ultrasound; MRI, magnetic resonance imaging.

^a^ Median and interquartile range.

Single IUFD occurred in 89.0% (95% CI 78.8–965.2) in the donor and in 11.1% (95% CI 3.8–21.2) in the recipient twin.

The results of the quality assessment of the included studies using the NOS are presented in Table 2. Most of the included studies had a moderate score for the selection, comparability, and outcome domains. The main weaknesses of the included studies were their retrospective design, small sample size, and heterogeneity of outcomes observed.

**TABLE 2:** Methodological assessment of included studies based on the Newcastle–Ottawa Score (NOS).

Author	Year	Selection	Comparability	Outcome
Van De Sande^18^	2025	★★★	★★	★★★
Birk^19^	2025	★★	★	★★
Tricca^20^	2024	★★	★★	★★
Mustafa^21^	2022	★★	★	★★
Rosen^22^	2022	★★	★★	★★
Jeong^15^	2022	★★	★	★★
Donepudi^23^	2016	★★	★	★★
Fisher^24^	2016	★★	★	★★
Veujoz^25^	2015	★★	★	★
Slaghekke^26^	2017	★★	★★	★★
Ruano^27^	2017	★★	★	★

### 3.2 Synthesis of results

**FIGURE 2:**
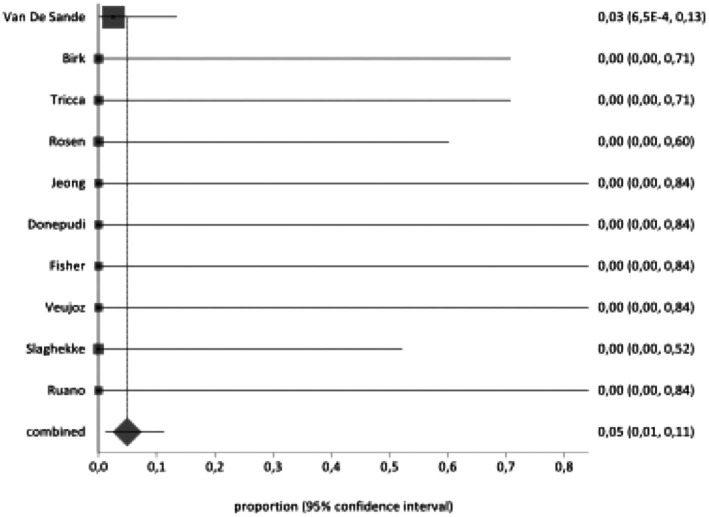
Pooled proportions for the occurrence of co‐twin death in monochorionic diamniotic pregnancies affected by twin anemia polycythemia sequence and complicated by single intrauterine fetal death.

Co‐twin IUFD occurred in 4.9% (95% CI 1.1–11.3; 1/64 fetuses) after a sIUFD in pregnancies affected by TAPS, while there was no case of NND (Figure 2). PTB, either spontaneous or iatrogenic, <34, 32, and 28 weeks of gestations occurred in 80.4% (95% CI 44.3–99.5; 5/6 patients), 80.4% (95% CI 44.3–99.5; 5/6 patients), and 50.0% (95% CI 16.3–83.7; 3/6 patients) of cases. IUT of the donor twin was required in 8.0% (95% CI 0.9–38.3). Cerebral anomalies at follow‐up fetal ultrasound or fetal MRI were reported in 15.0% (95% CI 8.2–78.3; 2/43 fetuses) and 11.4% (95% CI 2.4–55.7; 1/16 fetuses) of cases respectively, while anomalies at post‐natal imaging were reported in 9.4% (95% CI 5.6–57.6; 1/40 newborns) of cases. It was not possible to perform a pooled data synthesis on the occurrence of adverse neurological outcome because only the study by Van De Sande et al. explored this outcome and reported no case of adverse neurological event although the analysis was limited to the first day after birth. Likewise, none of the included studies reported on the occurrence of adverse long‐term neurodevelopmental outcome in the surviving fetus after sIUFD in TAPS.

Finally, it was not possible to perform a meaningful pooled data synthesis on the observed outcomes according to the type and staging of TAPS in view of the small number of included cases and even smaller number of events.

## 4 DISCUSSION

4.1

The findings from this systematic review showed that co‐twin death after sIUFD in MCDA pregnancies complicated by TAPS occurred in about 5% of cases, while there was no case of neonatal death. PTB, either spontaneous or iatrogenic, occurred in about 80% of cases <34 weeks and in about 50% of cases <28 weeks of gestation. The rate of cerebral anomalies detected either at follow‐up fetal ultrasound or fetal MRI was about 15%, while that at post‐natal imaging was 10%. Finally, the available data did not allow a comprehensive estimation of the rate of adverse neurological or neurodevelopmental outcome of these children.

4.2

This is, to the best of our knowledge, the first systematic review exploring the outcome of MCDA twin pregnancies affected by TAPS and complicated by sIUFD.

The primary strengths of this systematic review were the extensive collection of outcomes and comprehensive literature search, while its weaknesses included the small sample size in most of the included studies, which may have limited the analysis's power, their retrospective non‐randomized design, heterogeneity in outcome definitions, and the lack of standardized criteria for antenatal surveillance, intervention, time interval between diagnosis and intervention in case of post‐laser TAPS, and timing of delivery of MCDA affected by TAPS and complicated by sIUFD. Importantly, the results of this meta‐analysis were heavily influenced by the study by Van De Sande et al. which included the largest number of cases. Unfortunately, most included studies did not stratify their results according to the type (spontaneous vs. post‐laser), staging, and diagnostic criteria of TAPS, making subgroup analyses impossible. Notably, we could not determine the impact of prenatal interventions, primarily intra‐uterine blood transfusions, on the outcome of the surviving fetus after sIUFD in TAPS. Additionally, assessing the true risk of PTB was difficult because most studies did not report spontaneous and iatrogenic delivery rates separately, and this analysis was based on a limited number of cases. Finally, none of the included studies provided information on the long‐term neurological and neurodevelopmental outcomes of these children.

4.3

A systematic review by Mackie et al. reported a 40% rate of co‐twin IUFD and 28% of NND following sIUFD in MCDA twin pregnancies. PTB occurred in about 60% of cases, while abnormal pre‐ and post‐natal imaging following single demise was reported in 20% and 43% of the cases; neurodevelopmental morbidity affected 29% of the surviving children.^5^ Another systematic review by Giorgione et al. exploring the outcome of MCDA twin pregnancies complicated by TAPS reported that single and double IUFD occurred in 9.5% and 8.3% of cases with spontaneous and TAPS, and in 12.4% and 9.3% of those with post‐laser TAPS, although the authors did not specifically analyze the outcome of the surviving co‐twin after sIUFD.^28^

4.4

Management of sIUFD in MCDA twin pregnancies is challenging. The presence of inter‐twin placental anastomoses is responsible for an acute reverse transfusion from the deceased to the surviving twin, which occurs immediately after sIUFD, leading to severe hypotension and ischemia, which in turn are responsible for the high risk of death and neurological morbidities reported in these fetuses. Such damage is likely to occur immediately after the demise of one twin; thus, immediate delivery does not reduce the risk of death or abnormal outcome while there is still uncertainty on whether other interventions, such as intra‐uterine blood transfusion, may affect the outcome of the surviving twin after sIUFD.

Although inter‐twin placental anastomoses are present in every monochorionic placenta, several differences in the placental angioarchitecture among uncomplicated pregnancies and those with TTTS and TAPS exist and may account for a different risk of death or morbidity when an IUFD occurs.^29^

TAPS commonly shows a unique placental angioarchitecture characterized by a lower number of arterio‐venous anastomoses compared to uncomplicated pregnancies and those with TTTS, which also show a small (<1 mm) diameter.^30^ This peculiar placental angioarchitecture is responsible for the slow unidirectional passage of red blood cells from the donor twin to the recipient twin, gradually leading to discordant hemoglobin levels. The slowness of this process allows for hemodynamic compensation between the two circulations, which is hypothesized to be the reason for the absence of the amniotic fluid volume discordancy seen in classic TTTS. However, this chronic transfusion of red blood cells is also responsible for the high‐output cardiac failure of the donor twin, which accounts for the higher risk of neurodevelopmental delay compared to the recipient. Placentas of pregnancies affected by TAPS also show a reduced number and diameter of artero‐arterial anastomoses, which, together with veno‐venous anastomoses, mediates the acute blood transfusion from the deceased to the surviving twin when a sIUFD occur.^29^, ^30^ In this scenario, the small size and number of arterio‐arterial anastomoses may account for a lower risk of massive reverse transfusion, explaining the low rate of co‐twin death reported in our meta‐analysis.

Single IUFD in MCDA twin pregnancies has a profound impact on the risk of abnormal neurodevelopmental outcome. Cerebral hypoperfusion due to hypotension is responsible for brain ischemia, which affects mainly the peri‐ventricular area of the brain, which in turn is responsible for the high risk of neurodevelopmental delay reported in these children. In the present systematic review, the presence of brain anomalies detected exclusively at follow‐up fetal ultrasound, MRI, or at birth was reported in about 10–15% of cases. These figures are lower compared to those reported in previous studies not including TAPS and focusing mainly on apparently uncomplicated pregnancies or those affected by TTTS. It is plausible that the smaller diameter and number of placental anastomoses may be responsible for this lower risk of abnormal cerebral imaging. In this scenario, neurosonography or fetal MRI should be considered in order to identify cerebral lesions that can impact the prognosis (Table 3).

**TABLE 3:** Pooled proportions for the outcomes observed in the present systematic review.

Outcome	Studies (*n*)^Ref^	Cases (*n*/*N*)	Pooled proportions (95% CI)	*I* ^2^ (%)
Co‐twin IUFD	11^15^, ^18^, ^19^, ^20^, ^22^, ^23^, ^24^, ^25^, ^26^, ^27^	1/64	4.94 (1.12–11.26)	0
Co‐twin NND	5^18^, ^19^, ^20^, ^25^	0/47	0 (0–6.77)	0
PTB <34 weeks	2^19^, ^20^	5/6	80.44 (44.25–99.46)	7.4
PTB <32 weeks	2^19^, ^20^	5/6	80.44 (44.25–99.46)	7.4
PTB <28 weeks	2^19^, ^20^	3/6	50.00 (16.26–83.74)	0
Need for intra‐uterine transfusion	3^15^, ^18^, ^28^	1/44	8.00 (0.87–38.29)	59
Abnormal prenatal brain imaging	2^18^, ^22^	2/43	14.99 (8.23–78.28)	87.6
Anomalies detected at fetal MRI	2^18^, ^20^	1/16	11.37 (2.38–55.73)	66.6
Anomalies detected at post‐natal brain imaging	2^18^, ^20^	1/40	9.39 (5.61–57.63)	76.2

Importantly, about 8% of the surviving fetus showed signs of fetal anemia, requiring intra‐uterine transfusion. In this scenario, close ultrasound monitoring after the diagnosis of sIUFD is crucial to identify fetuses with anemia, which may benefit from intra‐uterine transfusion.

The risk of adverse neurodevelopmental outcome in pregnancies complicated by TAPS has not been extensively reported in the published literature. Tollenaar et al. reported that in a cohort of 98 children affected by TAPS, abnormal neurodevelopmental outcome was reported in 30 of TAPS survivors and was found more often in donors (44% vs. 18%).^31^ Importantly, donors demonstrated lower cognitive scores compared with recipients. Gestational age at delivery and the severity of fetal anemia were the only parameters independently associated with the risk of neurodevelopmental impairment at multivariate analysis.

Unfortunately, we could not perform a pooled data analysis on the risk of neurological and neurodevelopmental outcomes in the surviving fetus after sIUFD in MCDA twin pregnancies with TAPS. However, patients should be informed regarding the potential risk of long‐term neurocognitive developmental impairments and the need for long‐term neurological follow‐up.

## 5 CONCLUSION

The risk of co‐twin death after sIUFD in pregnancies complicated by TAPS is low, while that of PTB, either iatrogenic or spontaneous, remains high and similar to what is reported in MCDA twin pregnancies complicated by sIUFD. The surviving twin has a 15% risk of developing cerebral lesion, likely as a result of acute hypotension and ischemia following co‐twin death, thus highlighting the need for a detailed imaging assessment, either using ultrasound or MRI. Conversely, the findings from this systematic review highlight the lack of long‐term data on the neurodevelopmental outcome of the surviving twin after sIUFD in TAPS, highlighting the need for large studies looking at long‐term outcomes and sharing common protocols for prenatal management and postnatal assessment of these fetuses.

## AUTHOR CONTRIBUTIONS

Marina Piergianni: conceived the present idea, investigated, performed the analysis, drafted and revised the manuscript. Asma Khalil: supervised the findings of this work, revised the manuscript, discussed the results. Giuseppe Rizzo: supervised the findings of this work, revised the manuscript, and discussed the results. Ilenia Mappa: supervised the findings of this work, revised the manuscript. Lorenza Della Valle: investigator, performed the analysis, discussed the results. Hiba J Mustafa: supervised the findings of this work, revised the manuscript. Alberto Galindo: supervised the findings of this work, revised the manuscript. Alireza A Shamshisaz: supervised the findings of this work, revised the manuscript. Francesco D'Antonio: conceived the present idea, performed the analysis, supervised the findings of this work, drafted and revised the manuscript.

## CONFLICT OF INTEREST STATEMENT

No conflict of interest to declare by any of the authors.

## Supporting information


**Table S1.** Diagnostic criteria for TAPS in the included studies.

**Table S2.** Excluded studies and reason for the exclusion.

## Data Availability

The data that support the findings of this study are available from the corresponding author upon reasonable request.
