# Supplementary material for: Perinatal outcomes following single intrauterine death in monochorionic twin pregnancies complicated by twin anemia polycythemia sequence: Systematic review and meta‐analysis
Source: Acta Obstet Gynecol Scand. 2026 May 9;105(6):1017–25. doi: 10.1111/aogs.70189 (PMC13191807; doi:10.1111/aogs.70189)
Supplement: Supplementary file 1 — Table S1. Diagnostic criteria for TAPS in the included studies. [file AOGS-105-1017-s002.docx]

**Supplementary Table 1.** Diagnostic criteria for TAPS in the included studies.

| **Author** | **Year** | **Diagnostic criteria for TAPS** |
| --- | --- | --- |
| Van De Sande | 2025 | MCA PSV discrepancy between the twins, deﬁned as MCA-PSV > 1.5 multiples of the median (MoM) in the TAPS donor combined with MCA-PSV < 1.0 MoM in the TAPS recipient, in the absence of TOPS |
| Birk | 2025 | progressive and significant worsening of middle cerebral artery peak systolic velocity (MCA PSV) discordance after laser or persistence of > 0.5 multiples of the median (MoM) discordance 2 weeks post laser |
| Tricca | 2024 | Difference of delta MCA‐PSV > 0.5 MoM between the two fetuses according to the new classification system |
| Mustafa | 2022 | MCA‐PSV > 1.5 MoM in one twin and <1 MoM in the other twin, group B (delta Delphi): delta inter‐twin MCAPSV > 1 MoM, and group C (delta 0.5): delta inter‐twin MCAPSV > 0.5 MoM. |
| Rosen | 2022 | Measurement over 1.5 MOM in the TAPS donor, suggestive of fetal anaemia, combined with MCA‐PSV less than 1 MOM in the TAPS recipient, suggestive of fetal polycythemia, without signs of TTTS. |
| Jeong | 2022 | MCA-PSV of >1.5 MoM in the donor twin and <1.0 MoM in the recipient twin |
| Donepudi | 2016 | TAPS was deﬁned as discordance in the preoperative MCA-PSV, with one of the twin fetuses having MCA-PSV ≤ 1.0 MoM and the other having MCA-PSV ≥ 1.5 MoM 8 . |
| Fisher | 2016 | MCA-PSV >1.5 MoM in one twin and <1.0 MoM in the other and post-natally when Hb was <12 g/dL in the anaemic twin and >20 g/dL in the polycythaemic twin. |
| Veujoz | 2015 | MCA PSV > 1.5 MoM in one fetus that coincided with a decreased velocity of < 1.0 MoM in the cotwin, in the absence of TOPS. Diagnosis of postnatal TAPS was based on an intertwin haemoglobin difference of ≥ 8.0 g/dL and at least one of the following criteria: reticulocyte count ratio > 1.7 or small anastomoses (< 1 mm) at the placental surface. |
| Slaghekke | 2014 | MCA PSV > 1.5 MoM in one fetus that coincided with a decreased velocity of < 1.0 MoM in the cotwin, in the absence of TOPS. Diagnosis of postnatal TAPS was based on an intertwin haemoglobin difference of ≥ 8.0 g/dL and at least one of the following criteria: reticulocyte count ratio > 1.7 or small anastomoses (< 1 mm) at the placental surface. |
| Ruano | 2013 | MCA PSV >1.5 MoM in one twin (anemic fetus) and a PSV< 1.0 MoM in the polycythemic twin, in addition to the postnatal conﬁrmation of an intertwin haemoglobin difference of greater than 8 g/dL 15. |

MCA: middle cerebral artery; MoM: multiple of the medians. PSV: peak systolic velocity; TOPS: twin oligohydramnios polyhydramnios sequence.
