# Supplementary material for: Perinatal outcomes following single intrauterine death in monochorionic twin pregnancies complicated by twin anemia polycythemia sequence: Systematic review and meta‐analysis
Source: Acta Obstet Gynecol Scand. 2026 May 9;105(6):1017–25. doi: 10.1111/aogs.70189 (PMC13191807; doi:10.1111/aogs.70189)
Supplement: Supplementary file 2 — Table S2. Excluded studies and reason for the exclusion. [file AOGS-105-1017-s001.docx]

**Supplementary Table 2.** Excluded studies and reason for the exclusion.

| **Author** | **Year** | **Title** | **Reason for the exclusion** |
| --- | --- | --- | --- |
| Zhang | 2025 | Managing two cases of twin anemia–polycythemia sequence in monochorionic twin pregnancies | Only 2 cases of TAPS were included in this study, but none of them experienced IUD |
| Horie | 2025 | Rapid Change From Spontaneous Twin Anemia Polycythemia Sequence to Twin-to-Twin Transfusion Syndrome | Case Report |
| Van de Sande | 2025 | Doppler ultrasound changes in twin anemia polycythemia sequence treated with fetoscopic laser surgery | This study is likely to share cases with that of Van de Sande et al. |
| Kyvernitakis | 2024 | Stage-based recipient and donor outcome in twin-to-twin transfusion syndrome treated by fetoscopic laser surgery using Solomon technique | No data on the outcomes observed in the present systematic review could be extrapolated from this study |
| Munoz | 2024 | Perinatal outcomes of fetoscopic selective laser photocoagulation for spontaneous twin-anemia polycythemia sequence | Only 1 pregnancy complicated by single IUD was included in this series |
| Shantz | 2024 | Treatment of Twin Anemia Polycythemia Sequence with Donor Transfusion and Partial Recipient Exchange Transfusion: Procedural Considerations and Outcomes | No data on the outcomes observed in the present systematic review could be extrapolated from this study |
| Munoz | 2024 | Perinatal outcomes of fetoscopic selective laser photocoagulation for spontaneous twin-anemia polycythemia sequence | Only one case on IUD was included in this series |
| Harada | 2023 | Twin anemia polycythemia sequence in discordant dichorionic twins | Case report |
| De Sainte Fare | 2022 | The Value of Delta Middle Cerebral Artery Peak Systolic Velocity for the Prediction of Twin Anemia-Polycythemia Sequence—Analysis of a Heterogenous Cohort of Monochorionic Twins | No data on pregnancy outcome after sIUD |
| Hochberg | 2021 | Fetal and neonatal brain lesions following laser ablation for twin‐to‐twin transfusion syndrome as detected by pre and post-natal brain imaging | No data on the outcomes observed in the present systematic review could be extrapolated from this study |
| Mok | 2021 | Predicting Adverse Outcomes in Monochorionic-Diamniotic Twins: The Role of Intertwin Discrepancy in Middle Cerebral Artery Doppler Measurements and the Cerebroplacental Ratio | No data on the outcomes observed in the present systematic review could be extrapolated from this study |
| Badr | 2020 | Antenatal management and neonatal outcomes of monochorionic twin pregnancies in a tertiary teaching hospital: a 10-year review | Only 1 case of IUD in pregnancies with TAPS was included in this series |
| Han | 2020 | Short- and long-term outcomes of pretermspontaneous twin anemia-polycythemia sequence | 10 cases with fetal death in utero (one or both fetuses) were excluded |
| Wang | 2020 | Longer distance between umbilical cord insertions is associated with spontaneous twin anemia polycythemia sequence | No data on pregnancy outcome after sIUD |
| Liu | 2020 | Performance of Antenatal Diagnostic Criteria of Twin-Anemia-Polycythemia Sequence | No data on pregnancy outcome after sIUD |
| Tollenaar | 2020 | Post-Laser Twin Anemia Polycythemia Sequence: Diagnosis, Management, and Outcome in an International Cohort of 164 Cases | This study is likely to share cases with that of Van de Sande et al. |
| Tollenaar | 2020 | Treatment and outcome of 370 cases with spontaneous or post-laser twin anemia–polycythemia sequence managed in 17 fetal therapy centers | This study is likely to share cases with that of Van de Sande et al. |
| Tollenaar | 2020 | High risk of long-term neurodevelopmental impairment in donor twins with spontaneous twin anemia–polycythemia sequence | No data on the outcomes observed in the present systematic review could be extrapolated from this study. Furthermore, this study is likely to share cases with that of Van de Sande et al. |
| Tollenaar | 2020 | Prevalence of placental dichotomy, fetal cardiomegaly and starry-sky liver in twin anemia–polycythemia sequence | This study is likely to share cases with that of Van de Sande et al. |
| Tollenaar | 2020 | Spontaneous twin anemia polycythemia sequence: diagnosis, management, and outcome in an international cohort of 249 cases | This study is likely to share cases with that of Van de Sande et al. |
| Han | 2020 | Short- and long-term outcomes of preterm spontaneous twin anemia-polycythemia sequence | Cases experiencing IUD were excluded from the analysis |
| Tollenaar | 2019 | Improved prediction of twin anemia–polycythemia sequence by delta middle cerebral artery peak systolic velocity: new antenatal classiﬁcation system | No data on the outcomes observed in the present systematic review could be extrapolated from this study. Furthermore, this study is likely to share cases with that of Van de Sande et al. |
| Tollenaar | 2019 | Twin-Twin Transfusion Syndrome with Anemia-Polycythemia: Prevalence, Characteristics, and Outcome | This study is likely to share cases with that of Van de Sande et al. |
| Tavares de Sousa | 2019 | Role of fetal intertwin difference in middle cerebral artery peak systolic velocity in predicting neonatal twin anemiapolycythemia sequence | Pregnancies complicated by IUD excluded from the analysis |
| Bamberg | 2018 | Quantiﬁed discordant placental echogenicity in twin anemia–polycythemia sequence (TAPS) and middle cerebral artery peak systolic velocity | No data on the outcomes observed in the present systematic review could be extrapolated from this study |
| Tollenaar | 2017 | Can color difference on the maternal side of the placenta distinguish between acute peripartum twinetwin transfusion syndrome and twin anemiae polycythemia sequence? | No data on the outcomes observed in the present systematic review could be extrapolated from this study |
| Robinson | 2017 | Fetal brain injury in complicated monochorionic pregnancies: diagnostic yield of prenatal MRI following surveillance ultrasound and inﬂuence on prognostic counselling† | No case of IUD was reported in this study |
| Fishel-Bartal | 2016 | Can middle cerebral artery peak systolic velocity predict polycythemia in monochorionic–diamniotic twins? Evidence from a prospective cohort study | Cases experiencing IUD were excluded from the analysis |
| Stagnati | 2016 | Intertwin discrepancy in middle cerebral artery peak systolic velocity and third-trimester fetal growth restriction in monochorionic–diamniotic twin pregnancy | Cases affected by TAPS were excluded from the analysis |
| Hiersch | 2016 | Amniotic ﬂuid discordance in monochorionic diamniotic twin pregnancies is associated with increased risk for twin anemia–polycythemia sequence | There was no case of MCDA pregnancy affected by TAPS and complicated by IUFD |
| Ashwal | 2015 | Twin Anemia-Polycythemia Sequence:Perinatal Management and Outcome | IUD was an exclusion criterion for this study |
| Yokouchi | 2015 | Incidence of spontaneous twin anemia–polycythemia sequence in monochorionic–diamniotic twin pregnancies: Single-center prospective study | Pregnancies complicated by IUFD were excluded from this study |
| Slaghekke | 2015 | Middle cerebral artery peak systolic velocity to predict fetal hemoglobin levels in twin anemia–polycythemia sequence | It was not possible to extrapolate data on the outcome of pregnancies affected by TAPS and complicated by IUFD |
| Yokouchi | 2015 | Incidence of spontaneous twin anemia–polycythemia sequence in monochorionic–diamniotic twin pregnancies: Single-center prospective study | No case of IUD was reported in this study |
| Taniguchi | 2015 | Twin Anemia-Polycythemia Sequence after Laser Surgery for Twin-Twin Transfusion Syndrome and Maternal Morbidity | No case of IUD was reported in this study |
| Van Winden | 2015 | Pre-Operative Twin Anemia/Polycythemia in the Setting of Twin-Twin Transfusion Syndrome (TTTS) | No case of IUD was reported in this study |
| Abdel-Sattar | 2014 | Treatment of Complicated Spontaneous Twin Anemia-Polycythemia Sequence via Fetoscopic Laser Ablation of the Vascular Communications | Only 1 case of pregnancy affected by TAPS and complicated by IUD was included in this study |
| Mabuchi | 2014 | Clinical characteristics of monochorionic twins with large hemoglobin level discordance at birth | No case of IUD was reported in this study |
| Slaghekke | 2014 | Neurodevelopmental outcome in twin anemia–polycythemia sequence after laser surgery for twin–twin transfusion syndrome | No data on the outcomes observed in the present systematic review could be extrapolated from this study |
| Movva | 2014 | Discrepancy in placental echogenicity: a sign of twin anemia polycythemia sequence. | No data on the outcomes observed in the present systematic review could be extrapolated from this study |
| Rustico | 2013 | Fetal and Maternal Complications after Selective Fetoscopic Laser Surgery for Twin-to-Twin Transfusion Syndrome: A Single-Center Experience | Only 1 case of IUD in pregnancies with TAPS was included in this series |
| De Villiers | 2013 | Placental characteristics in monochorionic twins with spontaneous versus post-laser twin anemia-polycythemia sequence | No data on the outcomes observed in the present systematic review could be extrapolated from this study |
| Favre | 2013 | Vascular pattern in monochorionic placentas with spontaneous TAPS and TTTS with residual anastomoses after laser: a case–control study | No data on the outcomes observed in the present systematic review could be extrapolated from this study |
| Gucciardo | 2010 | Twin anemia polycythemia sequence from a prenatal perspective | Less than 2 cases of IUD were included in this study |
| Weingertner | 2010 | Clinical and placental characteristics in four new cases of twin anemia–polycythemia sequence | Less than 2 cases of IUD were included in this study |
| Chmait | 2010 | Residual Vascular Communications in Twin-twin Transfusion Syndrome Treated with Sequential Laser Surgery: Frequency and Clinical Implicationsq | No data on the outcomes observed in the present systematic review could be extrapolated from this study |
| Weingertner | 2010 | Clinical and placental characteristics in four new cases of twin anemia–polycythemia sequence | No data on the outcomes observed in the present systematic review could be extrapolated from this study |
